# Use of Antiepileptic Drugs for Hyperkinetic Movement Disorders

**DOI:** 10.2174/157015910793358187

**Published:** 2010-12

**Authors:** A Siniscalchi, L Gallelli, G De Sarro

**Affiliations:** 1 Department of Neuroscience, Neurology Division, Annunziata Hospital, Cosenza, Italy; 2 Pharmacology, Department of Experimental and Clinical Medicine, Faculty of Medicine, University Magna Graecia of Catanzaro, Clinical Pharmacology Unit, Mater Domini University Hospital, Catanzaro, Italy

**Keywords:** Antiepilteptic drugs, GABA, hyperkinetic movement disorders.

## Abstract

Many studies investigated the use of antiepileptic drugs (AEDs) in several neurological diseases other than epilepsy. These neurological disorders, usually, involve neuronal excitability through the modulating of ion channels, receptors and intracellular signaling pathways, and are the targets of the AEDs. This article provides a review of the clinical efficacy of both conventional and newer AEDs in hyperkinetic movement disorders. Some of these indications for AEDs have been established, while others are under investigation. The modulation of GABAergic transmission may explain the neuronal hyper-excitability that underlies some forms of hyperkinetic movement disorders. So, AEDs able to increase GABAergic neurotransmission may play a role in hyperkinetic movement disorders treatment. Therefore, AEDs could represent a useful therapeutic option in the management of hyperkinetic movement disorders where the available treatments are ineffective.

## INTRODUCTION

Antiepileptic drugs (AEDs), extensively used in the treatment of epilepsy, are usually classified into conventional and newer ones (see Table **1**).

The first group includes among others: bezodiazepines, carbamazepine, phenobarbital phenytoin and valproate, while the second one comprises: felbamate, gabapentin, lamotrigine, levetiracetam, oxcarbazepine, tiagabine, topiramate, vigabatrin and zonisamide [1-4]. Most of these drugs act *via* more than one mechanism of action, so AEDs may be divided into three principal groups: 1) blockers of voltage-dependent sodium and calcium channels, so they reduce high-frequency repetitive firing in neurons: carbamazepine, oxcarbazepine, gabapentin, lamotrigine, phenobarbital, phenytoin, topiramate, and valproate; 2) enhancers of GABA-mediated events (*via* the interaction with specific binding sites on the GABA-A receptor complex, the inhibition of GABA metabolism or the reduction of its neuronal uptake): benzodiazepine, gabapentin, phenobarbital, tiagabine, topiramate, vigabatrin, valproate; 3) blockers of the T-type calcium channels: ethosuximide and zonisamide [1-4]. An additional category of AEDs, comprising drugs which directly reduce the excitation mediated by ionotropic glutamate NMDA (i.e felbamate) and AMPA/kainite (i.e phenobarbital, topiramate) receptors, may be added [1, 3-6]. However, the exact mechanisms of action of the newer AEDs are not still fully clarified. 

Initial studies show the new agents to be equally efficacious but overall better tolerated than traditional agents. In addition, most of the new agents have a more favorable pharmacokinetic profile with limited hepatic metabolism, fewer drug interactions and adverse effects, i.e. cognitive impairment, hepatotoxicity and skin rash [2, 4, 7].

However, AEDs are extensively used to treat a wide range of disorders other than epilepsy, including both neurological and psychiatric conditions [4, 8]. The use of AEDs in other conditions is currently being explored [9].

The main pharmacodynamic mechanisms responsible for the clinical efficacy of AEDs in non-epileptic disorders include the modulation of both gamma-aminobutyric acidergic (GABAergic) or glutamatergic neurotransmission and the modulation of voltage-gated ion channels or intracellular signalling pathways [9, 10]. Current knowledge indicates that several AEDs have more than one mechanism of action, contributing to their therapeutic efficacy [10]. However, indirect mechanisms may be also involved, such as modulation of other neurotransmitters, including the monoamine. Conclusive evidences of the effects of AEDs on other neurotransmitter systems has not been published. One study describes the changes in monoamine and metabolites plasma levels during the treatment with lamotrigine, carbamazepine and phenytoin [11]. Valproate is one of the most thoroughly studied AEDs and its effects on other neurotransitters have been reviewed by Löscher [12]. Disordered function of GABAergic neurons in the basal ganglia may be responsible of several involuntary movements, the hallmarks of hyperkinetic disorders.

Hyperkinetic movement disorders include tremor, paroxysmal dyskinesias (dystonia and chorea), myoclonus, restless legs syndrome, hemifacial spasms and other disorders [13-16]. Pathophysiologically, these disorders are characterized by a reduced basal ganglia outpout resulting in a dis-inhibition of the thalamo-cortical systems, releasing cortical motor areas to allow movements that would normally be suppressed. Furthermore, the specific manifestations of the hyperkinetic disease may be determined by presence or absence of other abnormalities, such as in the patterning or the level of synchronization of basal ganglia output [17]. Evidences supported that the efficacy of AEDs in hyperkinetic movement disorders is still inadequate for many of these disorders, even if newer AEDs, which are more tolerability, are under investigations. Therefore, it is important to improve the clinical trials in larger numbers of patients with homogeneity of diagnosis, to establish the efficacy of the AEDs. 

The purpose of the present review is to relate the mechanisms of action of AEDs to pathophysiological mechanisms and clinical efficacy in hyperkinetic movement disorders (Table **2**).

## DRUGS TREATMENT OF HYPERKINETIC MOVEMENT DISORDERS

### 1 Essential Tremor

Essential tremor (ET) is a progressive neurological disorder characterized by a synchronous activation of antagonistic muscles [18] and it is defined as a rythmical, involuntary oscillatory movement induced by alternative contraction of agonist and antagonist muscles [8, 19]. ET may be caused by a deficiency in the α1-subunit of GABA-A receptor, as documented in a knockout model of mice [59]. This mechanism elucidates that a dysfunction in GABAergic system in the major motor pathway represents a potential target for pharmacotherapy, and benzodiazepines, which are GABA-A receptor agonists, may be efficacious in ET treatment [20, 21]. This animal model overlaps with ET in humans and shows a loss of inhibitory neurotransmission by cerebellar Purkînje cells, without the presence of Purkînje cells degeneration [19]. Moreover, it has been reported that diazepam had no effect on ET, whereas dizocilpine is able to reduce tremor, reflecting that functionality and pharmacological sensitivity may be altered in genetic models, and impairment of contributes to essential tremor [20, 21]. Based on their mechanisms of action, AEDs that enhance GABAergic neurotransmission could be effective in the treatment of ET. Drugs with this potential include conventional (primidone, clonazepam) and newer AEDs (tiagabine, gabapentin, pregabalin, topiramate, levetiracetam and zonisamide).

Primidone is the most effective drug used in the treatment of ET, even if it may be related with the development of toxic side-effects (e.g. nausea, vomiting, sedation, confusion, and ataxia) [22, 23]. The anti-tremor effects of primidone have been confirmed by several open trials and placebo-controlled studies and it is recommended (level A) by the American Academy of Neurology [23]. The efficacy of primidone in the management of ET is mostly attributed to the parent compound rather than to its metabolites phenylethyl-malonamide or phenobarbital. In some patients, primidone might be more efficacious than propranolol, and a combination of the two drugs might be more efficacious than either drug alone [22]. In addition to propanolol and primidone, the benzodiazepine drugs (such as clonazepam) could play a role in ET treatment [24]. The newer AEDs able to enhance the GABAergic neurotransmission, e.g. gabapentin, pregabalin, topiramate and possibly levetiracetam, may play a role in the treatment of neurological disorders such as ET [10]. In a clinical study Gironell *et al*. [25] showed that gabapentin had the same efficacy of propranolol and was better than placebo. In contrast, in another previous study, with a small number of patients, no statistical differences was documented between gabapentin and placebo [26]. Zesiewicz *et al*. 2007 [27] documented the efficacy of pregabalin in the management of ET, but recently in a clinical study this improvement has not been found, even if the adverse events (the most common were drowsiness and dizziness) were similar in frequency in both clinical studies [28]. The effect of topiramate in essential tremor has been reported in several trials [29-31]. This effect could be explained by the proposed mechanism of action of topiramate, such as the modulation of GABA-A receptor [10]. In an old man with ET unresponsive to primidone treatment, the combined therapy of topiramate and declorazepam induced a rapid improvement of symptoms supporting a synergic effect of both drugs [32]

The positive effect of zonisamide on ET has been well reported [27, 33] also in patients with associated head tremor [34]. Although modulation of voltage-gated ion channels represents the main mechanism of action of zonisamide, it is also able to enhances the GABA release, to downregulate the number of GABA reuptake transporter proteins and to upregulate the glutamate transporter EAAC-1 [8, 35]. The effect of levetiracetam in both ET and cerebellar tremor seems to be promising and needs to be investigated in larger patient groups [36-38]. The primary site of action of levetiracetam seems to be the synaptic vesicle protein 2C (SV2A) possibly inhibiting the neurotransmitters release. Levetiracetam is also able to enhance the chloride currents in GABA-A receptor [39, 40]. It has been suggested that exocytosis of the excitatory neurotransmitter glutamate is inhibited by levetiracetam, and this could explain its clinical efficacy in ET, since the enhancement of GABAergic neurotransmission and the decrease in glutamatergic neurotransmission seem to be advantageous to reduce the ET [20]. However, another one study in fifteen patients, failed to demonstrate the efficacy of levetiracetam [41]. Recently tiagabine, a centrally acting GABA reuptake inhibitor, has been reported to exacerbate ET [42] while an open label trial in 5 ET patients revealed both the lack of clinical efficacy and the higher adverse events [43].

### 2 Paroxysmal Dyskinesias (PKD) 

Paroxysmal dyskinesias are a heterogenous group of hyperkinetic movement disorders recurring in an episodic fashion [44, 45]. If sudden movements trigger the attacks, the term paroxysmal kinesigenic dyskinesia (PKD) is used [46]. The typical clinical picture in such cases is of brief attacks of chorea/dystonia (seconds to minutes) affecting a limb that are precipitated by sudden movements [47]. In most patients, one limb or side of the body is more frequently affected than the other, and the attacks are typically unilateral, even if generalized attacks are occasionally reported. This condition is usually responsive to AEDs [19, 45].

In fact, Chillag and Deroos in a retrospective study reported a complete resolution of idiopathic paroxysmal kinesigenic dyskinesia in four patients treated with lower-doses of oxcarbazepine monotherapy [48].

The pathophysiology of PKD is unclear at the time of this review. However, neuronal ion channelopathies have been documented in several paroxysmal movement disorders such as episodic ataxia type 1 (KCNA1) and type 2 (CANCA1) [12, 36, 49, 50] and in paroxysmal neurological disorders without associated movement disorders including familial hemiplegic migraine [51-54] and some forms of familial epilepsy [48, 55, 56]. 

Thus considering the paroxysmal character of the attacks, the presence of prodromic aura-like symptoms, the short time of the attacks and their response to AEDs [57, 58], it is hypothesized that PKD could be also related to ion channels disorders at subcortical levels. In agreement with this hypothesis, several reports documented the presence of people with different age-related expression affected by both PKD and epilepsy [33, 59]. On the other hand, the occurrence of dystonia in 70-80% of PKD episodes might indicate a pathophysiological process similar to primitive dystonia, where deficits in cortical, brainstem and spinal inhibitory circuits, due to disordered basal ganglia modulation of cortical motor output, have been detected. Evidence to support the role of the basal ganglia in the pathophysiology of PKD is the observation of secondary PKD in patients with focal basal ganglia lesions [60]. Recently, Fourcad *et al*. [61] in a study in 19 patients, supported the hypothesis that channel dysfunction and basal ganglia lesions may be together involved in the pathophysiology of PKD. Moreover, Huang and Hwang [62] described that a few epilepsy and paroxysmal movement disorders are "channelopathies", indicating that they may share some commons pathophysiological mechanisms and a possible "overlap".

#### 2.1 Dystonia

Dystonia is defined as a neurological disorder dominated by sustained muscle contractions, which frequently cause twisting, repetitive, and patterned movements or abnormal postures [63]. Dystonic movements can be slow, manifested by prolonged dystonic spasms resulting in abnormal postures, or can be rapid and jerk-like movements [19, 64]. Dystonia might also present as a rhythmical movement, most noticeable when the patient attempts to correct the underlying abnormal dystonic posture [19, 64]. This dystonic tremor can be abolished if the patient is asked to relax and allow the body part to move in the direction of the dystonic pull (null position) [19, 64]. 

The adult-onset idiopathic dystonias, the most common dystonic disorders seen in neurological practice, are usually focal and remain focal or segmental [17, 19, 64]. Examples include cranial dystonia (blepharospasm or oromandibular or lingual dystonia), cervical dystonia (spasmodic torticollis), laryngeal dystonia (spasmodic dysphonia), and dystonic writer’s cramp [22, 65]. Pharmacological treatment is largely empiric, rather than scientific. Attacks of kinesigenic paroxysmal dystonia can be controlled with anticonvulsants (carbamazepine, phenytoin, levetiracetam, topiramate and gabapentin) [19, 66-68]. The non- kinesigenic form of paroxysmal dystonia are less responsive to pharmacological treatment, even if clonazepam might be used [69].

#### 2.2 Chorea

Chorea consists of irregular, purposeless, abrupt, rapid, brief, jerky, unsustained movements that flow randomly from one part of the body to another [19, 65, 70].

The term “choreoathetosis” describes the combination of chorea and athetosis, a slow form of chorea manifested by writhing movements predominantly involving distal extremities [19, 65, 70]

Ballism, a severe form of chorea, comprises wide amplitude, flinging movements, usually involves the proximal limbs and most often affects only one side of the body (hemiballism) [19, 65, 70, 71].

Typically a lesion in the contralateral subthalamic nucleus is the cause of ballism, however, pathology in other subcortical areas could be also involved. Rarely, ballism can occur bilaterally (biballism or paraballism) [19, 65, 71]. Many patients with ballism have also distal choreic movements and, as recovery occurs, hemiballism often transform into hemichorea and hemidystonia [19, 65]. Chorea is defined as primary when it is idiopathic or genetic in origin or secondary when it is related to other causes (i.e. such as vascular, metabolic and immunologic diseases, infections and drugs) [70-72]. The pathophysiological processes for primary neurodegenerative chorea, such as Huntington's disease, clearly involve both excitotoxicity and the inhibition of histone acetylation, accompanied by the loss of GABAergic medium-sized spiny neurons in the striatum [71-73].

The AEDs can be efficacious in choreiform movements. In particular, valproate potentiates the GABA activity and modulates the potassium channels and could be also able to have direct membrane-stabilizing effects [74-76]. Recently, Zadori and co-workers [73] documented in an experimental model of Huntington's disease that chronic intraperitoneal administration of valproate ameliorates the survival and the motor performance. Therefore valproate’s action may be mediated by both GABAergic and antiexcitotoxic effects, but could be also related to the inhibition of histone deacetylase. Carbamazepine can also reduce the choreiform movements in some patients through the stabilization of inactivated state of voltage-gated sodium channels [74, 75, 77]. The newer AEDs, may represent an alternative in the treatment of paroxysmal kinesiogenic choreoathetosis; in fact, gabapentin and levetiracetam have been reported to be useful in the management of patient with hemichorea/hemiballism [78, 79], while topiramate is reported to improve both vascular hemichorea/hemiballism [80, 81] and vascular generalized chorea [55]. Moreover, recently Vlas and co-workers [50] described the efficacy of levetiracetam (start dose of 2.5mg⁄kg⁄day, titrated to a final dose of 10mg⁄kg⁄day) to reduce choreoathetosis in two patients with dyskinetic cerebral palsy, without the development of side effects.

### 3 Myoclonus

Myoclonus is defined as sudden jerks typically lasting 10 to 50 milliseconds, with the duration of movements rarely longer than 100 milliseconds [14]. Myoclonus is usually a positive phenomenon, causing synchronized muscle contractions in single or multiple muscle groups. Myoclonus jerks can be irregular, rhythmic, or even oscillatory, due to a dysfunction in cortical, brainstem, or spinal motor system. Neurodegenerative syndromes, encephalitis, and toxic-metabolic disorders responsible for cortical injuries may cause myoclonus with or without seizures. Myoclonus occurs as an ictal phenomenon in many epilepsy syndromes including idiopathic (e.g. juvenile myoclonic epilepsy of JME), symptomatic (e.g. myoclonic epilepsy or infancy) and progressive disorders (e.g. Lafora body disease).

Myoclonus may be induced by hyperexcitability of many neurons at several levels of the motor system as a result of neuronal injury or degenerative disorder. The dysfunction of inhibitory mechanisms may explain the neuronal hyperexcitability that underlies some forms of myoclonus. GABA mediates the majority of fast inhibitory synaptic transmission in the CNS; glycine is the inhibitory transmitter for some neurons in the brainstem and spinal cord [82]. The postsynaptic GABA and glycine receptors are pentameric arrangements of subunits around a central pore that conducts chloride when opened by transmitter binding, thereby inhibiting the postsynaptic cells. Bicuculline and picrotoxin, GABA-A receptor antagonists, can induce myoclonus [24, 82]. Animal models of myoclonus have been generated by producing alterations of postsynaptic GABA-A and glycine receptors [83]. Dysfunction of GABAergic system in the pre-motoneuronal circuitry may be responsible for the generation of spinal myoclonus. A previous study performed in a patient with rhythmic segmental myoclonus documented the presence of hyperactivity in the dorsal horn inhibitory interneurons [84]. However, drugs that increase GABAergic transmission, like clonazepam and valproate are the first-line treatments for myoclonus [82, 85]. Clonazepam is a direct agonist of the GABA-A receptor [82], while valproic acid is an indirect agonist and increases the brain GABA levels without interaction with the receptor [82]. In a clinical study the myoclonic hyperkinesias in Huntington’s disease patients showed a dose-depended improvement with valproate [85]. Moreover, on GABA-A receptor, levetiracetam is able to blocks the effects of the negative allosteric modulators (i.e. zinc) [86]. Previous studies indicate that levetiracetam is effective in the treatment of cortical and spinal myoclonus [87, 88]. Although a disorder of spinal inhibitory interneurons is the favored pathology in most cases of spinal myoclonus, histological evidence exists in which motoneurons are prime candidate [84]. The hyperexcitability of the motoneuron could be due to overactivity of postsynaptic excitatory neurotransmission or to changes in the distribution of voltage-gated channels in the membrane [89]. In agreement, we reported a case of a patient with segmental myoclonus in an amputation stump that ameliorated markedly after the treatment with topiramate, probably due to the reduction of motoneuronal hyperactivity through a decrease of glutamate function at postsynaptic sites or to inhibition of sodium and calcium currents [90]. However, this effect has not documented with gabapentin treatment. In fact, has been well reported that gabapentin at the dose from 600 mg to 1, 800 mg may increase the risk of development of de novo myoclonus or worsening of myoclonus in patients with preexistent myoclonus in about 2 weeks of treatment [91].

### 4 Restless Legs Syndrome

Restless legs syndrome (RLS) is a a sensorimotor disorder common in people over 65 years old and characterized by an irresistible urge to move the legs, accompanied by uncomfortable and unpleasant sensations that diminish with motor activity and worsen at rest [92]. These symptoms worsen in the evening and at night, leading to difficulty in sleeping and therefore can induce insomnia and depression. This syndrome can be a primary disorder or a secondary one, associated with for example iron-deficiency, uremia or polyneuropathies [92, 93]. 

The specific pathophysiology of idiopathic RLS is not well known but recent studies have raised the hypothesis of diencephalospinal pathway dysfunction. This pathway includes spinal, subcortical and cortical structures [94, 95].

Some patients (typically with painful symptoms) may be responsive to carbamazepine, gabapentin, valproic acid and benzodiazepines [36, 96]. Carbamazepine demonstrated its efficacy in relieving paresthesia in two placebo-controlled studies [4], but it is not recommended for occurrence of toxic side effects and drug interactions [97-99].

However, the efficacy of oxcarbazepine has recently been documented in three patients with RLS [100]. Gabapentin (dosage: 200 mg up to 2,000 mg, mean: 800-1,855 mg) could be efficacious for the treatment of RLS, but current evidence is limited to randomized double-blind controlled studies and two randomized controlled studies [96]. Valproate is likely efficacious for the treatment of RLS, even if the special monitoring is recommended for side effects, like hepatotoxicity [101].

Benzodiazepines, especially clonazepam, could be useful for intermittent RLS symptoms and because of their hypnotic effects they may be particularly useful to treat sleep onset insomnia caused by RLS [96, 102]. In this syndrome, topiramate is considered to be investigational [103]. However, at the moment, the use of AEDs remain the second-line option in the treatment of RLS.

### 5 Hemifacial Spasms

Hemifacial spasm (HFS) is a potentially disabling clinical disorder characterized by chronic twitching or spasm of on side of the facial muscles innervated by seventh cranial nerve [56]. The pathophysiological mechanisms for the genesis and maintenance of HFS have not yet been defined, however it results from vascular compression of the facial nerve at the root entry zone [56, 104]. The recent use of ADEs could be related to the reduction of the hyperexcitability of the nucleus of the seventh nerve. These effects may be mediated by 1) a block of voltage-sensitive sodium and T-type calcium channels, 2) a reduction of excitatory glutamatergic transmission and 3) an enhancement of GABAergic neurotransmission [3, 10].

There are only isolated reports of pain improvement in HFS patients using ADEs such as carbamazepine [105], clonazepam [106], felbamate [107], levetiracetam [108, 109], pregabalin [110], gabapentin [66, 111] and topiramate [112]. Moreover, we recently reported a patient with idiopathic HFS responsive to zonisamide-treatment [113].

## CONCLUSION AND DIRECTIONS FOR FUTURE RESEARCH

The primary indication for AEDs remains certainly epilepsy, even if other neurological conditions may be treated with these drugs when the typical treatments are ineffective. Several pathophysiological mechanisms inducing a neuronal excitability seems to be involved in an imbalance of both GABAergic and glutamatergic neurotransmissions and therefore could be similar in epilepsy and hyperkinetic movement disorders. The main targets for the action of the AEDs include enhancement of GABAergic inhibition, decreased glutamatergic excitation, modulation of voltage-gated sodium and calcium channels, and effects on intracellular signalling pathways. All of these mechanisms are of importance in controlling neuronal excitability in different ways. Future research on intracellular mechanisms might become decisive for a better understanting of the similarities between epilepsy and these disorders. Clinical observation, however, suggests that compared with conventional AEDs, the newer AEDs are more tolerated and have less pharmacodynamic interactions [7]. 

In the treatment of hyperkinetic movement disorders there is the potential to use pharmacological agents with GABA-potentiating properties, and GABAergic hypofunction in the basal ganglia is cited as an important mechanism underlying the pathophysiology of several hyperkinetic movement disorders. AEDs that seem to enhance GABAergic neurotransmission can be seen to have a role in these neurological disorders. AEDs with effects on decreasing glutamatergic neurotransmission on and/or voltage sodium or calcium channels may be advantageous in several hyper-kinetic movement disorders, like PKD, spinal myoclonus and HFS. Research supporting the efficacy of AEDs in the management of hyperkinetic movement disorders is still inadequate and there are only isolated reports regarding the role of AEDs in HFS patients. So, more prospective clinical trials are necessary to confirm the efficacy of both conventional and newer AEDs in hyperkinetic movement disorders and determine better the proportion of responders in a larger group of patients.

Current studies reported the clinical impact of pharmacogenetics on the pharmacological treatment of epilepsy. The genes may influence the outcome of drug treatment in individuals epileptic patients, in term of clinical efficacy and themselves may contribute to the development of side effects during treatment, that is the principal cause for dropped out of patients in clinical studies [114]. In fact, has been well reported that Asian patients HLA-B 1502 positive are at a higher risk for Stevens-Johnson syndrome during the treatment with carbamazepine [12].

Although many factors may contribute to variability of clinical outcome in individual patients, unpredictability differences in efficacy and adverse drug reactions, such as optimal doses of AEDs in patients with hyperkinetic movement disorders may be related to genetic variations. 

So, in the future, a better understanding of the AEDs mechanisms of actions in different hyperkinetic movement disorders should be provided by increased knowledge of the underlying molecular deficits in these disorders.

## Figures and Tables

**Table 1:** Classification of Antiepileptic Drugs

Conventional AEDs	Newer AEDs
Acetazolamide	Felbamate
Carbamazepine	Gabapentin
Clobazam	Lamotrigine
Clonazepam	Levetiracetam
Diazepam	Oxcarbazepine
Ethosuccimide	Pregabalin
Phenytoin	Tiagabine
Phenobarbital	Topiramate
Lorazepam	Vigabatrin
Piracetam	Zonisamide
Primidone	
Valproate	

**Table 2:** The Main Indication for Conventional and Newer AEDs in Hyperkinetic Movement Disorders. X= Effective Drug; 0=Not Effective Drug; ET= Essential Tremor; RLS= Restless Legs Syndrome; HFS= Hemifacial Spasms

Conventional Antiepiletic Drugs	ET	Dystonia	Choreoathetosis/Ballism	Myoclonus	RLS	HFS
Primidone	X (19, 23)					
Clonazepam	X (21)	X (19, 67-69)		X (82)		X (106)
Pnenytoin		X (19, 67, 68)				
Acid valproic			X (73-76)	X (82, 85)	X (4, 96, 101)	
Carbamazepine		X (19, 67, 68)	X (19, 75, 77)		X (4, 96)	X (105)
Newer Antiepiletic drugs						
Gabapentin	X/0 (8, 25)		X (79)		X (4, 96)	X (111)
Pregabalin	X/0 (28, 42)					X (110)
Felbamate						X (107)
Oxcarbazepine			X (48)		X (100)	
Topiramate	X (29-31, 38)	X (19, 67, 68)	X (80, 81, 96)	X (90)	X (103)	X (112)
Levetiracetam	X/0 (37-39, 41)	X (19, 67, 68)	X (50, 78)	X (87, 88)		X (108, 109)
Zonisamide	X (27, 33)					X (113)
